# Lithography-Free Planar Band-Pass Reflective Color Filter Using A Series Connection of Cavities

**DOI:** 10.1038/s41598-018-36540-8

**Published:** 2019-01-22

**Authors:** Amir Ghobadi, Hodjat Hajian, Mahmut Can Soydan, Bayram Butun, Ekmel Ozbay

**Affiliations:** 1 NANOTAM - Nanotechnology Research Center, Bilkent University, Ankara, 06800 Turkey; 2 Department of Electrical and Electronics Engineering, Bilkent University, Ankara, 06800 Turkey; 3 UNAM–National Nanotechnology Research Center, Bilkent University, Ankara, 06800 Turkey; 4 Institute of Materials Science and Nanotechnology, Bilkent University, Ankara, 06800 Turkey; 5 Department of Physics, Bilkent University, Ankara, 06800 Turkey

## Abstract

In this article, a lithography-free multilayer based color filter is realized using a proper series connection of two cavities that shows relatively high efficiency, high color purity, and a wide view angle. The proposed structure is a metal-insulator-metal-insulator-semiconductor (MIMIS) design. To optimize the device performance, at the first step, transfer matrix method (TMM) modeling is utilized to find the right choices of materials for each layer. Simulations are carried out later on to optimize the geometries of the layers to obtain our desired colors. Finally, the optimized devices are fabricated and experimentally characterized to evaluate our modelling findings. The characterization results of the fabricated samples prove the successful formation of efficient and wide view angle color filters. Unlike previously reported FP based designs that act as a band-stop filter in reflection mode (absorbing a narrow frequency range and reflecting the rest of the spectrum), this design generates a specific color by reflecting a narrow spectral range and absorbing the rest of the spectrum. The findings of this work can be extended to other multilayer structures where an efficient connection of cavities in a tandem scheme can propose functionalities that cannot be realized with conventional FP resonators.

## Introduction

Metamaterials and plasmonic nanostructures refer to a class of synthetic materials comprising designed inclusions that offer exotic properties^1^. Negative refraction^2^, artificial magnetism^3,4^, asymmetric transmission^5–7^, lasing^8,9^, cloak of invisibility^10,11^, photochemistry^12^ and sub-wavelength light confinement^13^ are examples of these attributes. The concept of light confinement by sub-wavelength geometries has been one of the most intensively studied areas in recent years. The use of metal-insulator (MI) based nanostructures can introduce a variety of functionalities spanning from ultra-broadband^14–24^ to ultra-narrowband perfect light absorbers^25–29^. Color generation, utilizing sub wavelength nanostructured geometries, has been the subject of many studies in this field^30–35^. These ultrathin structures are promising alternatives for conventional dye and colorant pigment-based filters that suffer from long-term stability due to their organic nature. Color filters can have potential applications, such as imaging, sensing, digital displays, and solar cells^34,36–41^. Up to now, different design structures have been employed to realize color filters in reflection and transmission modes. The use of different design structures based on surface plasmon resonance^32,33,42–47^, guided-mode resonance^48–52^, and photonic crystal^53–55^ and other innovative ideas^56–60^ are some of these approaches. While some of the above-mentioned proposed designs have high transmission/reflection efficiencies and high color purity, most of them operate in specific incident light polarization and angles. Therefore, the angle dependent functionality of these designs restricts their practical usage. Moreover, their main common bottleneck is the fabrication complexity. These periodic nano-patterned structures are fabricated by the use of electron-beam lithography (EBL) which this in turn limits their compatibility for upscaling. Therefore, in recent years, many studies were devoted to design planar lithography-free metal-insulator (MI) multilayer-based designs to generate different colors in reflection and transmission type color filters. This category of color filters is based upon Fabry-Perot (FP) resonances in a thin-film structure comprising metallic mirrors separated by an optically transparent insulator (or a highly absorbing semiconductor) medium^58,60–74^.

In a common FP resonance-based structure that is made of a metal-insulator-metal (MIM) stack, tuning the insulator layer can offer the narrowband reflection/transmission spectra in the entire visible light regime. In these FP resonators, the red-green-blue (RGB) additive colors and their complementary subtractive ones of cyan-magenta-yellow (CMY) can be obtained in transmission and reflection modes, respectively. Besides their simple, large scale compatible, and EBL free fabrication route, these structures can provide relatively high efficiency, high color purity, and low crosstalk. However, under oblique angle light incidence, the increase in optical passage length of light within the cavity imposes a blue shift in the resonance frequency of the design. This angular dependency is more pronounced in reflection mode, due to the longer optical path of the light. To mitigate this deficiency, several different methodologies have been conducted. The angle insensitivity response of the resonance can be attained by increasing the refractive index (n) and reducing the cavity thickness. This material can be a lossless material, such as Si_3_N_4_^55^, ZnS^69^, WO_3_^71^, and TiO_2_^61,64,66,68^, or a lossy material such as amorphous Silicon (a-Si)^68,70,72,73^. In the case of a lossy medium such as the cavity layer, the phase acquired upon the reflection at the metal (air)-semiconductor interface can be much greater than *π* and, therefore, unlike the lossless layers, the FP resonance can be attained in our desired wavelength in much thinner thicknesses^30,70^. Consequently, the optical path length difference would be negligible between normal and oblique incident lights. However, the drawback with this architecture is the fact that the cavity semiconductor layer can absorb light and reduce the efficiency of the color filter. This reduction is especially significant in the shorter wavelengths where the extinction coefficient (κ) of the semiconductor is larger. To lessen this adverse effect, a phase compensating dielectric overlay can be placed above the cavity to achieve the angle-insensitive characteristics without sacrificing the amplitude for all the primary reflective and transmissive colors^66,68^. However, adding this overlay on top of the FP design increases the bandwidth (BW) which this, in turn, leads to reduction in the color purity. Moreover, in all of these lithography-free FP designs, the structure performs as a band-stop filter at the reflection mode that absorbs a narrow frequency range and reflects the rest of the spectrum. Therefore, the generation of RGB additive colors is not possible in these designs and they can create these colors in the transmission spectra. But due to the inherent loss of the materials, transmissive color filters are in general less efficient compared to that of reflective ones.

In this study, a planar, large scale compatible and lithography-free reflection type RGB color filter with high efficiency, low color crosstalk, and wide viewing angle is fabricated. The proposed structure is a metal-insulator-metal-insulator-semiconductor (MIMIS) multilayer structure made of a series connection of a MIM and metal-insulator-semiconductor (MIS) cavities where the metal, insulator, and semiconductor layers are aluminum (Al), aluminum oxide (Al_2_O_3_)/zinc oxide (ZnO), and germanium (Ge). In this MIMIS architecture, the MIM design acts as a partial reflecting mirror that absorbs a portion of the visible light and reflects the rest of the spectrum. This reflected light experiences a new cavity resonance in the upper MIS configuration. As the consequence of this double resonance behavior, a narrow wavelength range of the spectrum would be reflected. By tuning the bottom insulator layer thickness, all of the visible colors have been realized in this reflection type color filter. Unlike conventional metal-insulator and metal-semiconductor based FP color filters that act as band-stop filters in the reflection mode (and generate subtractive CMY colors), this design operates as a band-pass filter to generate additive RGB colors. In the first step, the proposed design is fabricated using an alumina insulator layer. Later on, to improve the angular independency of the MIMIS structure, the Al_2_O_3_ layer is replaced with ZnO that has a higher index of refraction. The characterization results of the fabricated samples prove that the fabricated color filters have reflection peaks with amplitudes above 0.6 and high angular tolerance in which the change in the angle of incident light from 0° to 60° imposes a wavelength shift as small as 43 nm, 24 nm, and 14 nm for red, green, and blue colors at the p polarized light. These values are found to be 49 nm, 32 nm, and 25 nm for the s polarization case. It has been proved that the fabricated designs can offer high efficiency, low crosstalk, and nearly angle insensitive color filtering performance. This is the first report that demonstrates the generation of RGB colors in the reflection mode from a simple Fabry-Perot (FP) design which is required for several applications where a specific frequency range should be extracted from the incident white light. The findings of this study can be applied to another type of multilayer functional designs where an efficient connection of cavities in a tandem scheme can propose functionalities that cannot been realized with conventional FP resonators.

## Methods

### Device Fabrication

To fabricate this structure, first, the Si wafer is diced into small pieces. Later on, these samples are placed inside a Piranha solution for a duration of 10 min. Finally, hydrofluoric (HF) is utilized to remove the native oxide layer. The thermal evaporator machine is utilized to coat the Al and Ge layers at our desired thicknesses using the thermal evaporation technique. The deposition rate is fixed at 1 A°/sec and the chamber pressure is kept below 5 × 10^−6^ Torr throughout the deposition process. Atomic layer deposition (ALD) tool (Cambridge Nanotech Savannah S100) is employed to coat the Al_2_O_3_/ZnO layers in our desired thickness in a temperature of 250 °C. For this aim, Trimethylaluminum Al(CH_3_)_3_/Diethylzinc (C_2_H_5_)_2_Zn solutions are used as the deposition precursors for Al and Zn atoms and water as the oxygen source. The pulsing time is set at 15 ms and the purge duration is chosen as 10 s. The base pressure of the chamber is kept at 1 × 10^−2^ Torr during the process. The N_2_ flow is also fixed at 20 sccm.

### Simulations and Measurements

Optical simulations are conducted using a commercial finite-difference time-domain (FDTD) software package (Lumerical FDTD Solutions)^75^. For this aim, three dimensional (3D) simulations are employed. A plane-wave excitation in our desire wavelength range (380–800 nm) is used. The boundary conditions in the lateral directions (x and y) are chosen as periodic while these conditions are set as a perfectly matched layer (PML) for the z direction. To measure the normal incidence light reflection from the structure, we used an in-house setup that is made of a halogen lump as the incident light source where this source is integrated into to a microscope to collect the reflected light from the surface. This collected light intensity in the microscope is entered into a spectrometer (Newport OSM2). In all of the measurements, the obtained reflection values are normalized with the reflection data from a thick Al coated sample (which has near 100% reflection in our desired frequency range). Finally, a personal computer (PC) is utilized to extract and monitor the data. In addition, the reflection of the MIMIS samples at different light incident angles is measured utilizing a spectroscopic ellipsometer tool (J. A. Woollam Co. Inc. V-VASE) at two different S and P polarizations. The light angle of incidence is chosen as 30°, 45°, and 60°.

## Results and Discussion

**Figure 1:**
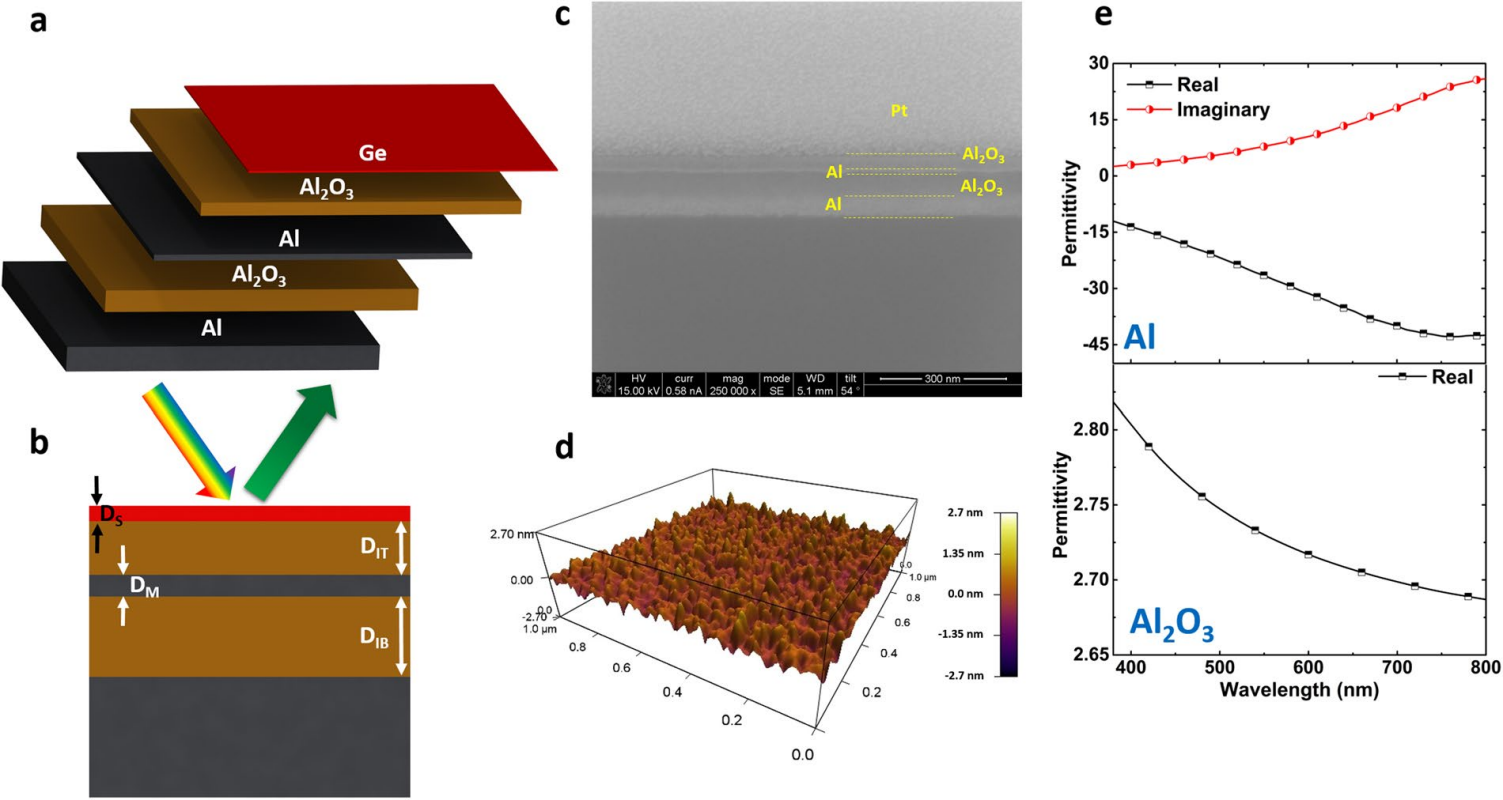
(**a**) The schematic representation of the MIMIS design and (**b**) its reflection upon excitation with a broadband light. (**c**) Cross sectional SEM image of the fabricated structure showing the successful formation of different layers. The proposed structure has a thick Al layer that reflects light back into the cavity, an Al_2_O_3_ layer that defines the spectral position of the reflection peak, a thin Al layer, a top Al_2_O_3_ insulator layer and top semiconductor Ge layer (**d**) the AFM image of the top Ge coating. The color bar spans from −2.7 nm to 2.7 nm. As we can see from this map, the top Ge layer has quite low roughness. (**e**) The experimentally extracted permittivity values for Al and Al_2_O_3_ layers in the range of 380–800 nm.

Figure 1a,b schematically represent the structure of the MIMIS multilayer design. In this configuration, the structure is made of; (1) a bottom 70 nm thick metal layer that is an optically thick Al coating acting as a reflecting mirror with no transmission throughout our desired frequency range, (2) an insulating separator having a thickness of *D*_*IB*_ that tailors the spectral position of the cavity resonance, (3) a thin partially reflecting/transmitting metal layer with a thickness of *D*_*M*_ to make a MIM cavity, (4) a top insulator layer of *D*_*IT*_, and (5) an ultrathin Ge semiconductor layer to create the MIS cavity resonance mode. The optimized designs have been fabricated as described in the methods section. As shown in Fig. 1c, the proposed stack formation is confirmed by looking at its scanning electron microscope (SEM) image. During sample milling (by focused ion beam (FIB)) to create a perpendicular side wall, a thin protective Pt is coated on sample. To capture the SEM image, the FIB is used to prepare a cross section of the sample. Therefore, considering the ultrathin thickness of the Ge (in the order of couple of nanometers), it is not possible to distinguish this layer in the SEM image. To evaluate the layer roughness and top Ge layer thickness, the atomic force microscope (AFM) measurement has been carried out. As can be seen in Fig. 1d, the coated Ge layer has a relatively smooth layer with an RMS roughness value of approx. 0.4 nm. Finally, spectroscopic ellipsometer measurement is used to extract the permittivity values of the deposited Al and Al_2_O_3_ layers in our desired range, see Fig. 1e.

**Figure 2:**
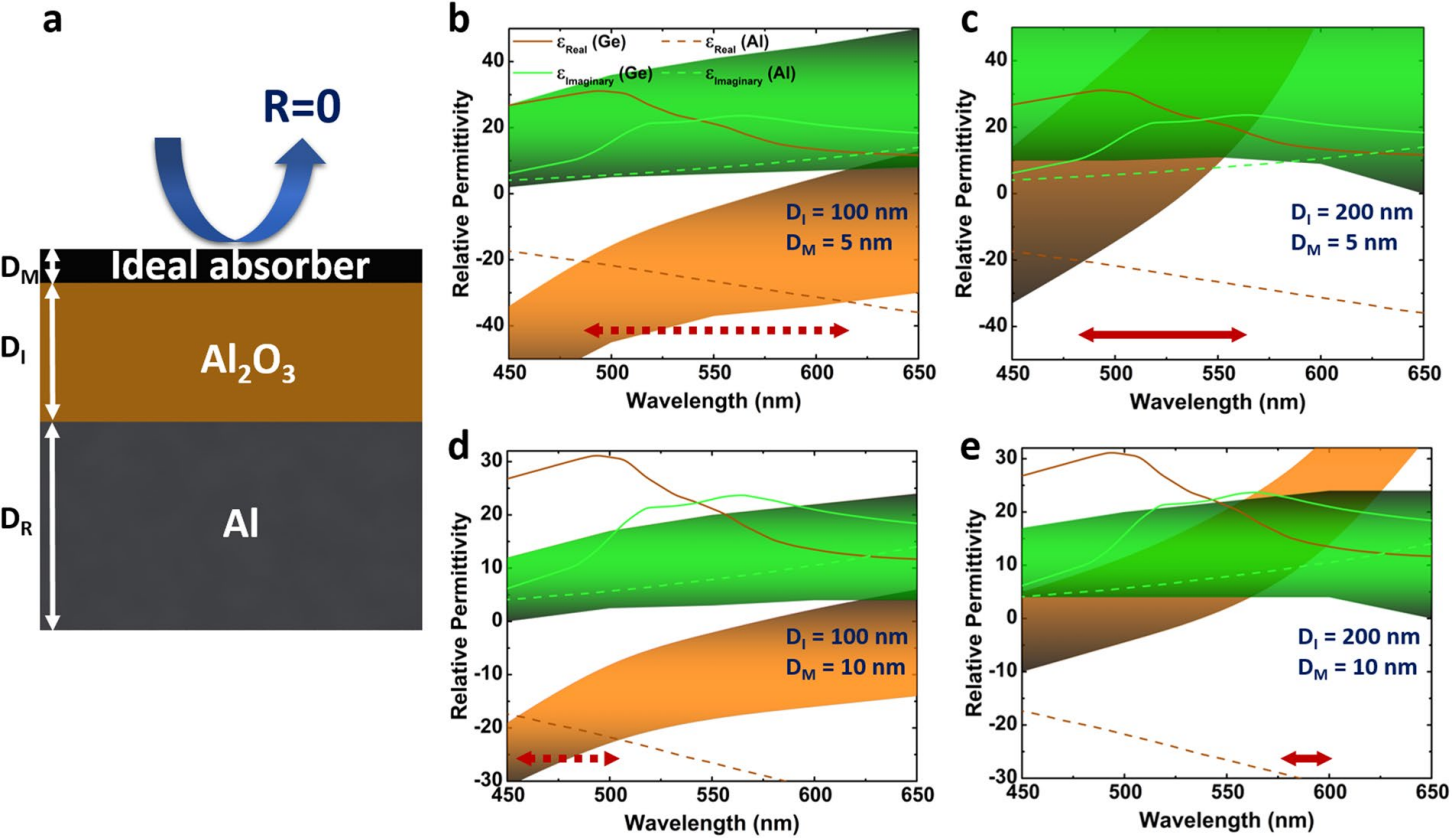
(**a**) The schematic representation of the cavity structure used in TMM model to find the ideal absorber case. The dimensions of the structure have been shown using vertical arrows. The bottom metal layer has a thickness of *D*_*R*_ = 70 nm, the spacer Al_2_O_3_ layer has a thickness of *D*_*I*_ and top ideal material has a thickness of *D*_*M*_. The ideal acceptable/tolerable regimes for real and imaginary portions of the permittivity to obtain an absorption above 0.8 for four different cases of (**b**) *D*_*I*_ = 100 nm, *D*_*M*_ = 5 nm, (**c**) *D*_*I*_ = 200 nm, *D*_*M*_ = 5 nm, (**d**) *D*_*I*_ = 100 nm, *D*_*M*_ = 10 nm, (**e**) *D*_*I*_ = 200 nm, *D*_*M*_ = 10 nm and their comparison with the permittivity values of Al and Ge. In these panels, the brown and green highlighted regions depict the tolerable region for real and imaginary parts of permittivity to obtain a reflection R below 0.1. If the permittivity of a material stays inside these regions, the material can absorb above 0.9 of the incident light. The red arrows show the spectral ranges that have this matching.

To begin with, it is essential to find the ideal materials for each layer of the stack. For this aim, the transfer matrix method (TMM) modeling approach is adopted to find the ideal top layer for the MIM and MIS cavities, as shown in Fig. 2a. In this model, Al and Al_2_O_3_ coatings are chosen as thick bottom reflector and separator, respectively. The ideal permittivity values to get a reflection below 0.2 is obtained for four different cases of *D*_*I*_ = 100 nm, 200 nm, and *D*_*M*_ = 5 nm, 10 nm. The thickness of the bottom Al mirror is set at 100 nm to ensure that light cannot pass across the layer throughout our desired frequency range. The details of the modelling are explained in our previous study^21^ and summarized in the following formulas. In this method, we suppose the MIM (or MIS) stack is surrounded with air having a permittivity of *ε*_*A*_. For p-polarized light, H_y_ is considered as:

1 $${H}_{y}(z)=\{\begin{array}{c}{A}_{i}{e}^{i{k}_{A}(z-{D}_{M})}+{A}_{r}{e}^{-i{k}_{A}(z-{D}_{M})},\,\,z > {D}_{M}\\ {M}_{11}{e}^{i{k}_{M}z}+{M}_{12}{e}^{-i{k}_{M}z},\,\,0 < z < {D}_{M}\\ {D}_{1}{e}^{i{k}_{I}z}+{D}_{2}{e}^{-i{k}_{I}z},\,\,-{D}_{I} < z < 0\\ {M}_{21}{e}^{i{k}_{R}(z+{D}_{I})}+{M}_{22}{e}^{-i{k}_{R}(z+{D}_{I})},\,\,-\,{D}_{I}-{D}_{R} < z < -\,{D}_{I}\\ {S}_{t}{e}^{i{k}_{S}[z+({D}_{I}+{D}_{R})]},\,\,z < -\,{D}_{I}-{D}_{R}\end{array}\}$$

and by adjusting proper boundary conditions, the amount of reflected wave is calculated as *R* = |*F*_12_/*F*_11_|^2^. Here, $$\,F=[\begin{array}{c}{F}_{11}\\ {F}_{12}\end{array}]={a}^{-1}{m}_{11}{m}_{12}^{-1}{d}_{1}{d}_{2}^{-1}{m}_{21}{m}_{22}^{-1}s$$ where:

2a $$a=[\begin{array}{cc}1 & 1\\ i{k}_{A}/{\varepsilon }_{A} & -i{k}_{A}/{\varepsilon }_{A}\end{array}],\,s=[\begin{array}{c}1\\ i{k}_{S}/{\varepsilon }_{S}\end{array}],$$

2b $$\begin{array}{rcl}{m}_{11} & = & [\begin{array}{cc}1 & 1\\ i{k}_{M}/{\varepsilon }_{M} & -i{k}_{M}/{\varepsilon }_{M}\end{array}],\\ {m}_{12} & = & [\begin{array}{cc}{e}^{i{k}_{M}{D}_{M}} & {e}^{-i{k}_{M}{D}_{M}}\\ i{k}_{M}{e}^{i{k}_{M}{D}_{M}}/{\varepsilon }_{M} & -i{k}_{M}{e}^{-i{k}_{M}{D}_{M}}/{\varepsilon }_{M}\end{array}],\end{array}$$

2c $$\begin{array}{rcl}{d}_{1} & = & [\begin{array}{cc}1 & 1\\ i{k}_{I}/{\varepsilon }_{I} & -i{k}_{I}/{\varepsilon }_{I}\end{array}],\\ {d}_{2} & = & [\begin{array}{cc}{e}^{i{k}_{I}{D}_{I}} & {e}^{-i{k}_{I}{D}_{I}}\\ i{k}_{I}{e}^{i{k}_{I}{D}_{I}}/{\varepsilon }_{I} & -i{k}_{I}{e}^{-i{k}_{I}{D}_{I}}/{\varepsilon }_{I}\end{array}],\end{array}$$

2d $$\begin{array}{rcl}{m}_{21} & = & [\begin{array}{cc}1 & 1\\ i{k}_{R}/{\varepsilon }_{R} & -i{k}_{R}/{\varepsilon }_{R}\end{array}],\\ {m}_{22} & = & [\begin{array}{cc}{e}^{i{k}_{R}{D}_{R}} & {e}^{-i{k}_{R}{D}_{R}}\\ i{k}_{R}{e}^{i{k}_{R}{D}_{R}}/{\varepsilon }_{R} & -i{k}_{R}{e}^{-i{k}_{R}{D}_{R}}/{\varepsilon }_{R}\end{array}],\end{array}$$

and ${k}_{i=({\rm{A}},{\rm{M}},{\rm{I}},R,S)}=\sqrt{{\varepsilon }_{i}{\omega }^{2}/{c}^{2}-{k}_{x}^{2}}$ which *c* is the speed of light in vacuum. Moreover, *D*_*I*_, *D*_*M*_, and *D*_*R*_ are the insulator, thin top coating and bottom thick coatings, respectively. The brown and green highlighted areas depict the tolerable region for these four different cases, as shown in Fig. 2. In other words, if a material’s permittivity values are located inside these regions in a specific wavelength range, the cavity can absorb above 80 percent of light. As already explained in the introduction section, our ultimate goal is to design a series connection of two cavities to get light reflection in a narrow wavelength range. Therefore, these two cavities should absorb shorter and longer wavelengths. The above part is an MIS cavity that is responsible for light absorption in shorter wavelengths and should pass the longer wavelengths. The below MIM cavity should absorb the longer wavelengths and reflect the shorter ones^58–60^. Therefore, the semiconductor layer should have a relatively weak absorption in the visible light region and metal layer should be a good reflector. Therefore, we have compared the permittivity values of Al and Ge layers with the ideal model. Figure 2b–e show the matching condition of these materials with the ideally tolerable region for four different cases. As can be clearly seen in this figure, at a thin insulator layer thickness of 100 nm, both the real and imaginary parts of Al permittivity can stay inside the region in specific wavelength ranges but there is no matching for Ge in this configuration. However, Ge shows a resonant light absorption in the case of *D*_*I*_ = 20 nm. Moreover, based on these results, as material thickness increases from 5 nm to 10 nm, the absorption BW gets narrower and shifts to other wavelength values. Therefore, both of these materials can act as a nearly perfect absorbers in our desired wavelength range and we only need to optimize the configuration to achieve this goal.

**Figure 3:**
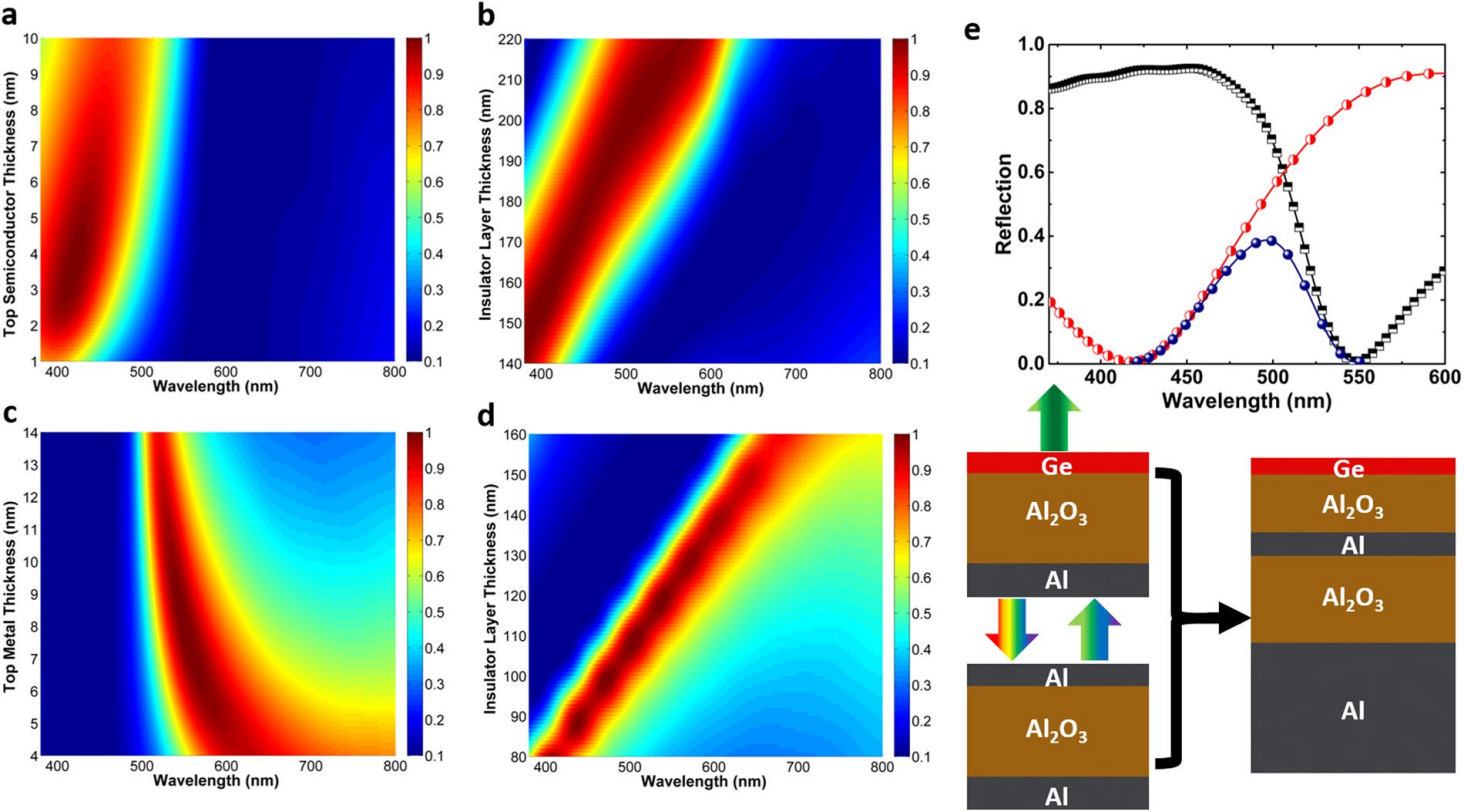
The contour plots of the absorption spectra for different (**a**) *D*_*M*_ and (**b**) *D*_*I*_ values in the Al-Al_2_O_3_-Ge MIS cavity. Increase in top insulator layer thickness (*D*_*M*_) makes the absorption strength less and the bandwidth wider. The optimum value is chosen as 3 nm. In this sweep the insulator layer thickness is fixed at 160 nm. By setting the top layer thickness at 3 nm, in the second counter plot, the insulator layer thickness is varied from 140 nm to 220 nm. This plot shows that the spectral position of light perfect absorption gets a red shift as we increase the insulator layer thickness. The absorption contour plot for different (**a**) *D*_*M*_ and (**b**) *D*_*I*_ values in the Al-Al_2_O_3_-Al MIM cavity. Similar to MIS case, we first sweep top metal layer thickness while fixing the insulator layer thickness in 100 nm. Considering both absorption strength and bandwidth the optimum top layer thickness is chosen as 9 nm. As shown in the other contour plot, the perfect absorption peak can be tuned by changing the insulator layer thickness. (**e**) The reflection spectra of the MIM (black), MIS (red), and MIMIS (blue) samples. As we can see from the schematic, the MIM structure absorbs the longer visible wavelengths while MIS cavity harvest the shorter wavelengths. In the spectral range in between these two absorption peaks both MIM and MIS cavities are inactive and light reflection occurs. Therefore, series connection these two cavities (MIMIS) could lead to a narrow light reflection.

At the first step, we need to find the optimal design geometries for both MIM and MIS cavities. To achieve this goal, numerical simulations are conducted using Lumerical FDTD Solutions^75^. The structures are excited with a plane wave in our frequency range of interest which is 380–800 nm. To record the reflection profile (R), a 2D power monitor is placed above the plane wave excitation. Moreover, the absorption spectra (A) of these samples is calculated using *A* = 1 − *R* formula. For the permittivity values of Al and Al_2_O_3_ layers, the experimental data (as shown in Fig. 1e) are employed. For the permittivity of the Ge layer, the Palik’s dispersive material data is utilized^76^. Initially, it is required to find the proper geometries for the MIS cavity. To achieve this, first the thickness of insulating coating is set at a specific value and top material thickness is swept to get the optimum geometries for near unity light absorption (which corresponds to a reflection near zero at that wavelength). Figure 3a shows the absorption contour plot for different *D*_*M*_ values in the Al-Al_2_O_3_-Ge MIS cavity configuration in which the thickness of insulator separator (*D*_*I*_) is fixed at 160 nm. As already explained, our MIMIS color filter works based on the subtraction of two resonance modes of MIM and MIS cavities. Therefore, the absorption spectra of the structure should have a sharp and nearly perfect peak in its resonance condition. Taking this into account, the optimum case is recorded for the Ge thickness of 3 nm. To evaluate the tunability of this resonance, this time, we fixed the Ge thickness at 3 nm and applied a sweep on the insulator layer thickness. Figure 3b proves that the nearly perfect light absorption (an absorption above 0.9) characteristic of the MIS design is retained and shifted toward longer wavelengths as the insulator layer thickness increases. Moreover, in line with modeling estimations, the spectral BW of the absorption response gets also wider. Similar to this case, the same route is followed to find the optimized geometries of the MIM configuration where the insulator layer thickness is fixed at 100 nm. For the Al layer, the best result is attained for a thickness of 9 nm and this nearly zero reflection functionality of the cavity design is kept for the thicker insulator layers, see Fig. 3c,d. All of the above-mentioned results demonstrate that the series connection of these two cavities can provide a reflection type color filter across the whole visible frequency range where the position of the reflection peak is located at the frequency that both of the cavities are inactive. This has been schematically shown in Fig. 3e. When light impinges on the sample surface, the larger wavelength values are trapped inside the bottom MIM cavity. In fact, this MIM cavity acts as a partial reflecting mirror that sends back a portion of the spectral region and absorbs the rest. The reflected portion creates a cavity with the above Ge layer and this time the shorter wavelengths would be absorbed. Thus, at the final output, a narrow band-pass reflection type color filter will be made. Unlike conventional MIM and MIS designs that perform as a reflective band-stop filter, this multilayer design operates as a band-pass filter which is necessary for several applications where reflection of a specific color and rejection of rest of the band is needed.

**Figure 4:**
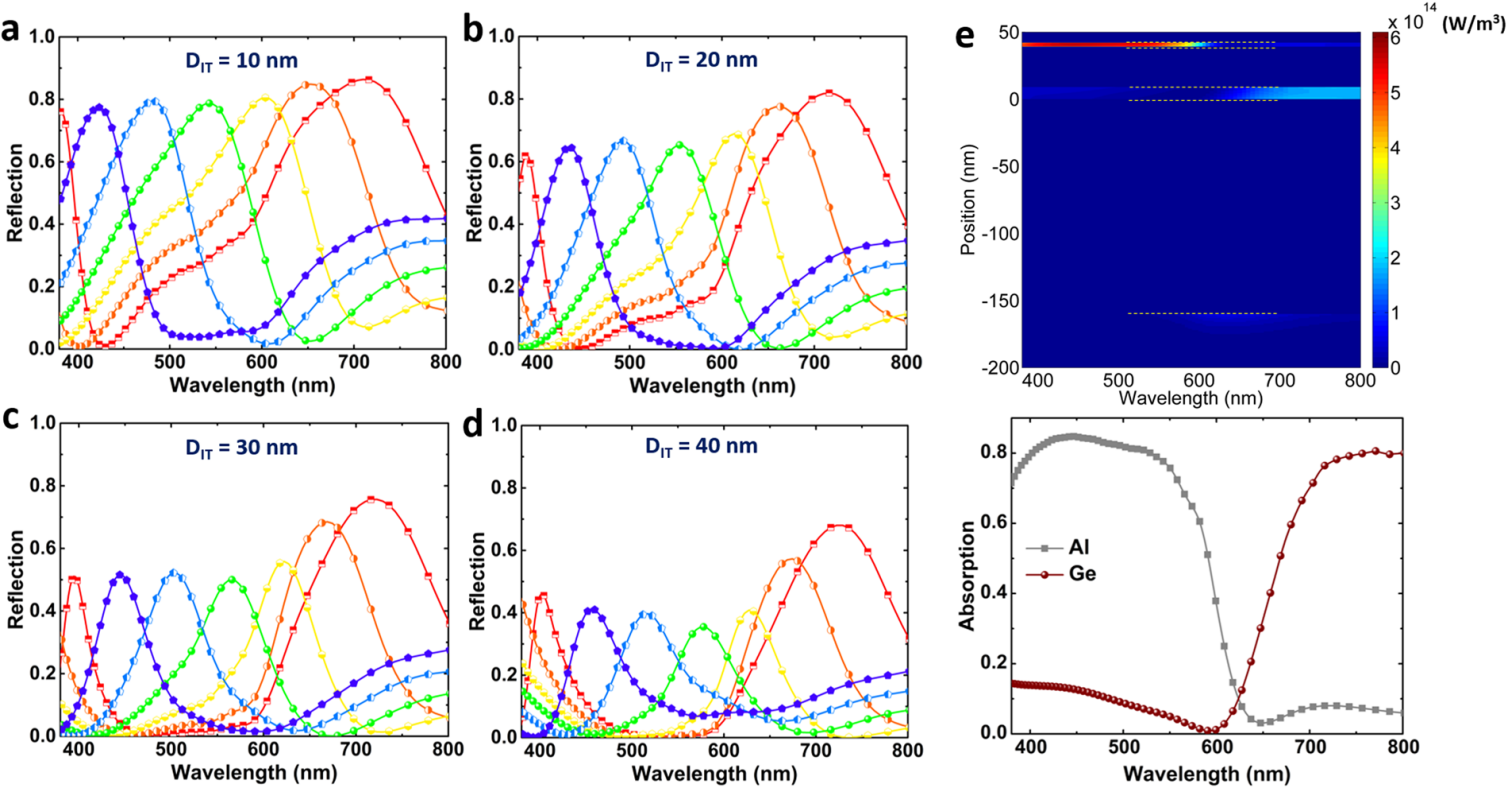
The reflection spectra of the MIMIS stack for different *D*_*IT*_ values of (**a**) 10 nm, (**b**) 20 nm, (**c**) 30 nm, and (**d**) 40 nm. In all of these cases, the thickness of bottom insulator is swept from 100 nm to 200 nm with a step size of 20 nm. In these simulations, the bottom metal layer, the middle Al layer and top Ge layer thicknesses have been fixed at optimal values of 70 nm, 9 nm, and 3 nm. (**e**) The contour plot showing the absorbed power across the MIMIS cavity as a function of incident light wavelength for the case of *D*_*IT*_ = 30 *nm* and *D*_*IB*_ = 160 *nm*. As we can see the absorption in lower wavelengths occurs at top Ge layer while longer wavelengths are mainly harvested in the middle Al layer. The linear graph also illustrates the amount of absorbed power in the Al and Ge layers.

In the next step, we need to scrutinize the role of different geometries on the absorption capability of the MIMIS stack. From the previous section, the optimal thickness values of Ge and middle Al coatings are fixed at 3 nm and 9 nm. The only geometries that can tune the spectral position and amplitude of the reflection peak are bottom and top insulators that are called *D*_*IB*_ and *D*_*IT*_, respectively. To gain insight on the impact of these two values on the reflection spectra of the MIMIS structure, we swept the *D*_*IB*_ values for different fixed *D*_*IT*_ amounts of 10 nm, 20 nm, 30 nm, and 40 nm. In each of these cases, the *D*_*IB*_ has been swept from 100 nm to 200 nm with a step size of 20 nm. Figure 4a–d illustrates the reflection spectra of the MIMIS design for different insulator layers thicknesses. As we can clearly notice, the thinner *D*_*IT*_ makes the reflection peak stronger but the full width at half maximum (FWHM) gets wider as well. As the thickness moves toward larger values, the amplitude decreases and the color purity (which is a factor effected by the peak BW) increases. Therefore, there is a trade-off between these two factors and *D*_*IT*_ is the geometry to provide an optimum condition for MIMIS design performance. Moreover, as already estimated from the modeling results, the bottom dielectric has the role to modulate the spectral position of the peak where an increase in its thickness induces a red shift in the peak position. Taking a look at the simulation results for *D*_*IT*_ values smaller than 30 nm, one can see that for the peaks resonating at orange and red frequencies, a small tail is extended toward shorter wavelengths which reduces the color purity of the design. The color purity is related to the resonance width of the reflection peak. Therefore, the optimum choice of *D*_*IT*_ thickness, which can simultaneously satisfy both color efficiency and purity, is 30 nm. To have a clear vision on the operation principle of this design, we have plotted the absorbed power profile inside this multilayer cavity as a function of incident light wavelength for the case of *D*_*IT*_ = 30 nm, and *D*_*IB*_ = 160 nm. Figure 4e shows that, at the shorter wavelengths, the upmost Ge layer is the active part of the MIMIS design while the middle thin Al coating gets activated at the longer wavelengths. The absorption inside Ge and Al layers have been monitored and depicted in this figure, as well.

**Figure 5:**
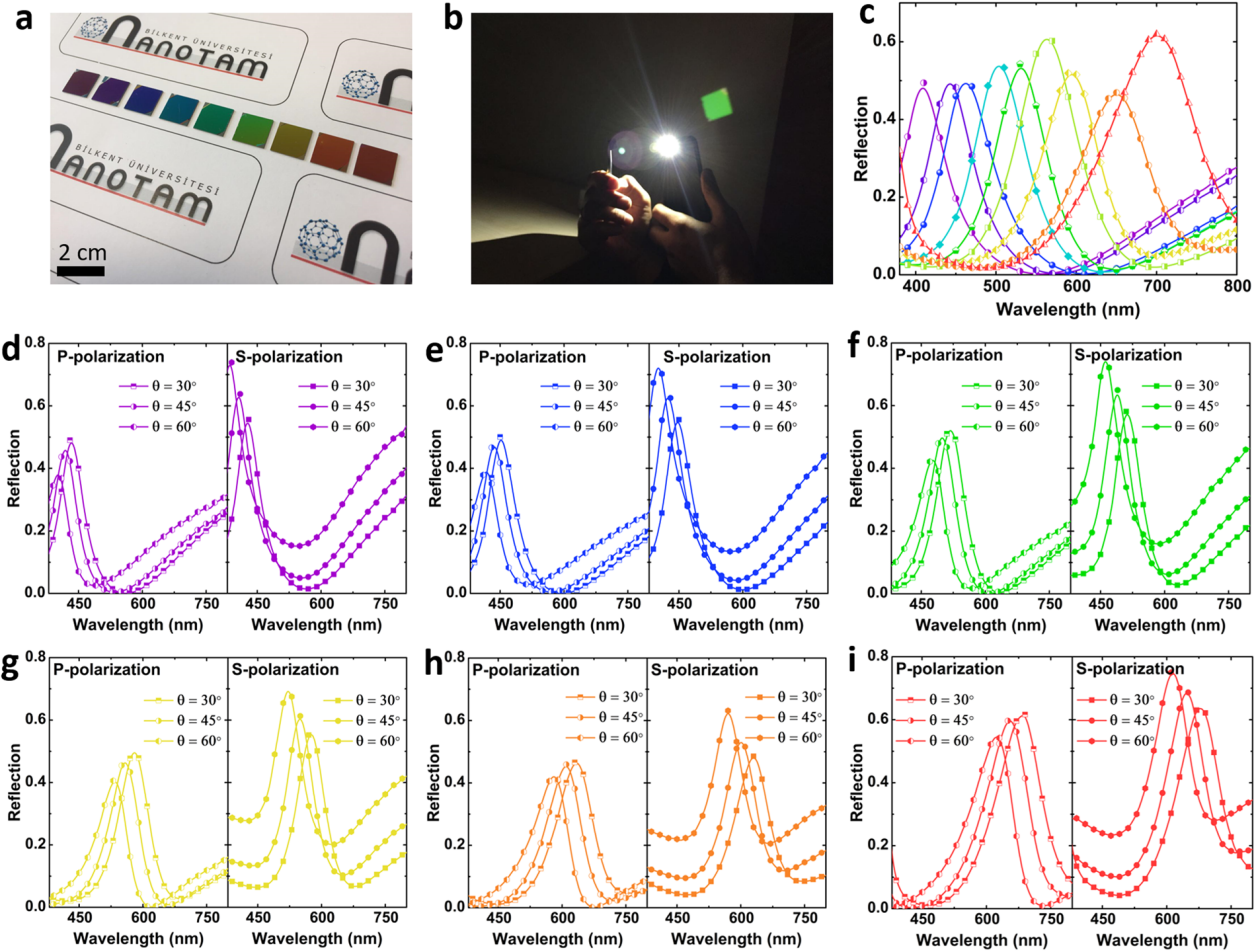
(**a**) The optical image of the fabricated MIMIS designs operating in different wavelengths in the visible regime. In all samples, the bottom metal layer thickness is 70 nm, the middle Al layer is 9 nm thick, the top insulator layer is 30 nm and top Ge layer is 3 nm. The bottom insulator layer thicknesses are 90 nm, 100 nm, 110 nm, 120 nm, 130 nm, 140 nm, 150 nm, 180 nm, and 195 nm. (**b**) The reflected green color from the fabricated sample upon excitation with white light. (**c**) The reflection spectra of different colored samples at normal light incidence. These data have been obtained using a reference sample of thick Al mirror as our 100% reflector. The angular response of (**d**) purple, (**e**) blue, (**f**) green, (**g**) yellow, (**h**) orange, and (**i**) red colored filters under different incidence angles (i.e. 30°, 45°, and 60°) and polarizations (S and P). In all samples, the reflection gets a blue shift as we go toward wider viewing angles.

To figure out the validity of our numerical results, we have fabricated and characterized different MIMIS samples with the obtained optimal values of 3 nm, 30 nm, and 9 nm for Ge, top insulator, and middle Al layers, respectively. The bottom insulator layer (*D*_*IB*_) is chosen at different values to generate different colors from ultraviolet (UV) to near infrared (NIR) regime, spanning the whole visible light colors. Figure 5a shows the optical image of the fabricated samples. In these samples, the *D*_*IB*_ is selected as 90 nm, 100 nm, 110 nm, 120 nm, 130 nm, 140 nm, 150 nm, 180 nm, and 195 nm for all different colored samples. The proposed architecture operates as a band-pass color filter that reflects a desired wavelength range of the incident white light, see Fig. 5b for the green light color filter that reflects green color from the white incident light. For the optical characterization of the MIMIS multilayer stack, the reflection profiles of the samples are recorded at normal light incidence using an in-house reflection measurement setup. Figure 5c shows the normal incident reflection spectra of different colored MIMIS samples. These results prove that the proposed structure can perform in a good trade-off between color efficiency and purity where the reflection has retained its efficiency relatively unchanged for all of the different colors. As explained in the methods section, we have used a planar thick Al layer as the reference for 100% light reflection. These findings demonstrate that this design principle, which operates based on the series connection of two cavities with two different absorption spectral positions, can provide a simple, EBL-free and up scaling compatible approach to fabricate efficient reflective RGB color filters. In addition, unlike other reported FP based multilayer designs that absorb a narrow frequency range and reflects the rest of the spectrum, this architecture can offer a narrow band-pass filter to reflect a specific color and absorb the remaining region. Another parameter that defines the practical applicability of a color filter is its angular response under oblique light illuminations. Figure 5d–i show the angular response of six different colors of violet, blue, green, yellow, orange, and red for two different P and S light polarizations. The light angles of incidence have been chosen as *θ* = 30°, 45°, 60°. As it can be clearly seen, for all the samples, the peak position experiences a blue shift for both polarizations. As the incident light angle gets wider, due to light refraction from different interfaces, the optical path of the light gets larger. Therefore, in all the cases, a blue shift is recorded for the samples upon shining them with an angled light source. Moreover, in this planar design, different Fresnel reflection coefficients for TE and TM polarizations lead to different angular responses. For instance, the blue color filter, resonating at 462 nm for normal incident light, moves its peak to 417 nm, and 401 nm (equivalent to a wavelength difference of Δ*λ* = 45 nm and 61 nm) under an incident angle of 60° for p and s polarizations, respectively. The amount of this shift gets larger as we move to a red color device. For the red color (with a peak reflection at *λ*_0_ = 700 nm), the amount of resonance spectral shift for p and s polarizations are 77 nm and 86 nm, respectively. To have a better qualitative comparison, the ratio of Δ*λ*_0_/*λ*_0_ is compared for three main colors of blue, green, and red. For p polarized incident light, these ratios are 0.09, 0.1, and 0.11 while this amount is increased to 0.13, 0.13, and 0.12 for s polarized light. Therefore, the color of the MIMIS structure is not retained in wide view angles which this, in turn, limits its applicability for practical applications.

**Figure 6:**
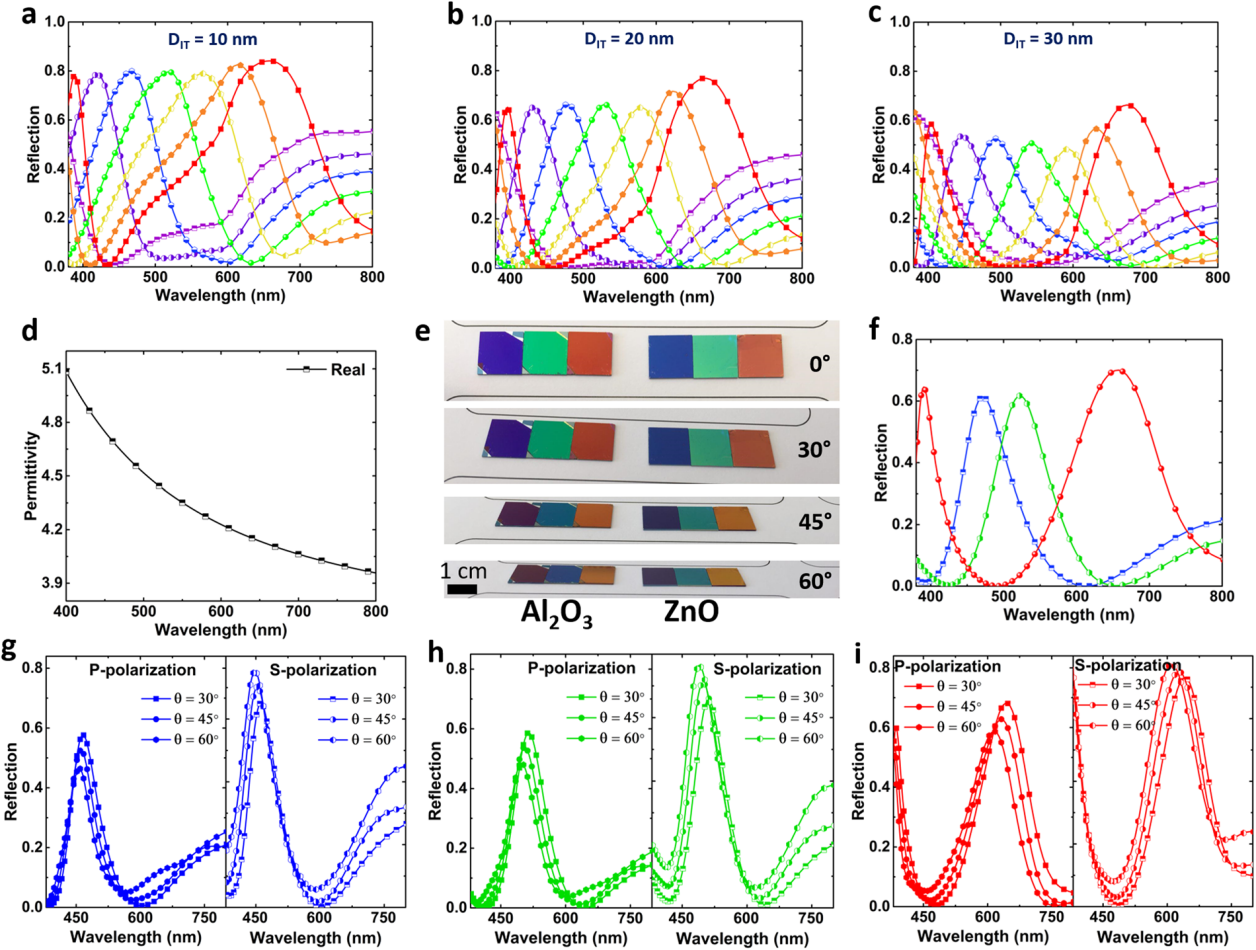
(**a**) The simulated reflection spectra of the ZnO based MIMIS stack for different *D*_*IT*_ values of (**a**) 10 nm, (**b**) 20 nm, (**c**) 30 nm. In all the simulations the bottom metal, middle metal, and Ge top layer thicknesses have been fixed at 70 nm, 9 nm, and 3 nm. The bottom insulator layer thickness is swept from 55 nm to 145 nm with a step size of 15 nm. (**d**) The experimentally extracted permittivity values of ZnO layer. (**e**) The comparison of the angular reflection response of the fabricated Al_2_O_3_ and ZnO based MIMIS designs. For this aim, a simple white light source was used, and the images are collected at 0°, 30°, 45°, and 60°. (**f**) The measured reflection response of RGB color filter samples at normal light incidence. In these designs, the top insulator layer thickness is fixed at 20 nm. The bottom insulator layer is chosen as 85 nm (blue), 100 nm (green), and 145 nm (red) to obtain all three main colors. The angular response of (**g**) blue, (**h**) green, (**i**) red color filters under different incidence angles and polarizations. In all of the samples, going toward wider view angles, the absorption peak experiences a blue shift.

It has been demonstrated earlier that a higher refractive index material can be utilized to reduce the angular dependence of the color filters^30,66,68,69^. Therefore, this time, Al_2_O_3_ is replaced with ZnO layer that has a higher light refraction index. In the first step, similar to the previous case, the optimum geometries for top and bottom insulators should be selected. Figure 6a–c illustrates the reflection of the ZnO based MIMIS design for three different *D*_*IT*_ values of 10 nm, 20 nm, and 30 nm. For each case, the bottom ZnO layer thickness is swept from 55 nm to 145 nm with a step size of 15 nm. It is worth noting that the experimentally extracted permittivity values of the ZnO layer (as shown in Fig. 6d) have been utilized in all the simulations. According to these results, this time the optimal case can be chosen as *D*_*IT*_ = 20 nm where a fine trade-off between reflection amplitude and its FWHM can be recorded for all the colors. Moreover, the reflection values are larger compared to that of Al_2_O_3_. Therefore, employing a high index insulator layer can provide a higher color efficiency. To evaluate our numerical findings, the optimal MIMIS designs are fabricated and optically characterized in our desired frequency range for three main colors of red, green, and blue. The bottom insulator layer thickness is picked as 85 nm, 100 nm, and 145 nm which corresponds to blue (*λ*_0_ = 471 nm), green (*λ*_0_ = 521 nm), and red (*λ*_0_ = 657 nm) colors. It should be noted that the Ge and middle Al layers thicknesses are similar to the previous design. Figure 6e compares the optical image of the fabricated samples for two different cases of Al_2_O_3_ and ZnO based MIMIS configurations. As can be clearly realized, ZnO based samples have a much better tolerance for oblique angle light incidence where even in extreme view angles they have a slight change in their color. As explained in the previous studies^30^, the variation of the resonance wavelength is inversely proportional to the refractive index of the dielectric layer in a metal-insulator pair based design. Therefore a high-index spacer layer can provide a better angular performance. In other words, as the refractive index of the insulator layer increases, based on Snell’s law, the refracted light gets closer to 90° and, therefore, the path difference between different angles becomes smaller. That is why ZnO has a better angular response compared to Al_2_O_3_. To evaluate this property, the normal incident reflection spectra of the samples are recorded for these three cases, as shown in Fig. 6f. This panel proves that the fabricated samples have a reflection amplitude higher than 0.6 with a relatively narrow FWHM. The angular responses of these three samples are also plotted at Fig. 6g–i. Similar to previous measurement, these spectra have been collected for both p and s polarizations. These results prove that the use of ZnO has significantly improved the angular response of the MIMIS cavity. Under incident angle of 60° for p polarized light, the shift in the resonance wavelength are Δ*λ*_0_ = 1 nm, 24 nm, and 43 nm for blue, green, and red colors, respectively. These values are found to be 25 nm, 32 nm, and 49 nm for s polarization case. The resonance wavelength change ratio ((Δ*λ*_0_)/*λ*_0_) can be also obtained for these samples to be able to have a qualitative comparison with previous samples. For the blue color, these ratios are 0.02 and 0.05 for p and s polarizations, respectively. For the red color reflecting sample, these values are found to be as 0.06 and 0.07 which are almost half of the ones obtained for the Al_2_O_3_ based MIMIS sample. Therefore, the utilization of a high index dielectric can simultaneously improve both efficiency and angular insensitivity of the samples. This work shows the extraordinary function of the tandem shaped cavities. As our future work, this design can be modified in a way that higher color efficiencies can be obtained. Moreover, using a simple fabrication technique, we can make spatially variant filters. In this case, sample is placed inside the chamber in a way that one part of it is close to the source and the other corner is far. By using this fabrication approach, a gradient insulator layer thickness is made. Thus, this in turn leads to monolithically integrated spatially variant color filters.

## Conclusion

In summary, for the first time in the literature, this work presents an efficient design strategy based on an MIMIS configuration to obtain RGB band-pass color generation at the reflection mode that cannot be attained with a conventional FP resonator. For this purpose, at the initial step, the optimal materials and geometries for each of the layers are found. Then, the proposed optimal design is fabricated and characterized for different colors spanning the whole visible region. Finally, to improve the angular response of the system, the dielectric medium is replaced with a higher refractive index layer. The final results demonstrate the formation of high efficiency RGB colors with a reflection above 0.6 and a wide view angle where the color is retained almost unaltered in wide view angles. Under an incident angle of 60° for p polarized light, the shifts in the resonance wavelength are Δ*λ*_0_ = 1 nm, 24 nm, and 43 nm for blue, green, and red. The resonance wavelength change ratio ((Δ*λ*_0_)/*λ*_0_) for the blue color device was found to be 0.02 and 0.05 for p and s polarizations, respectively. For the red sample, these values were 0.06 and 0.07, which demonstrates the efficient operation of these samples. The overall structure is a planar and EBL-free one which can be produced by simple preparation routes. The obtained results of this paper can be extended to other type of FP resonators where a proper connection of cavities can propose functionalities that cannot be attained in the conventional single cavity designs.
